# Developmental Trajectories of Regulating Attentional Selection Over Time

**DOI:** 10.3389/fpsyg.2012.00277

**Published:** 2012-08-13

**Authors:** Sabine Heim, Andreas Keil

**Affiliations:** ^1^Infancy Studies Laboratory, Center for Molecular and Behavioral Neuroscience, Rutgers The State University of New JerseyNewark, NJ, USA; ^2^Psychophysiology Laboratory, Department of Psychology, Center for the Study of Emotion and Attention, University of FloridaGainesville, FL, USA

**Keywords:** S, attentional control, emotion distraction, academic competency, rapid serial visual processing, attentional blink

## Abstract

Adaptive behavior in learning environments requires both the maintenance of an attentional focus on a task-set and suppression of distracting stimuli. This may be especially difficult when the competing information is more appealing than the target event. The aptitude to “pay attention” and resist distraction has often been noted as an important prerequisite of successful acquisition of intellectual abilities in children. This focused review draws on research that highlights interindividual differences in the temporal dynamics of attentional engagement and disengagement under competition, and their relation with age and cognitive/academic skills. Although basic strategies of attention control are present in very young children, the more refined ability to manage attentional resources over time in an economic and adaptive fashion appears during early school years, dramatically improves until the early teen years, and continues to develop into late adolescence. Across studies, parameters of attention control over time predict specific aspects of academic performance, rather than general intellectual ability. We conclude that the ability to strategically regulate the dynamic allocation of attention at rapid rates may represent an important element of cognitive and academic development.

## Conceptual Background: Attention and Performance

At the turn of the twentieth century, French psychologist Alfred Binet conducted a series of experiments on the development of sensory perception, attention, and intellectual ability, studying his two daughters Madeleine and Alice, then approximately 4 and 2.5 years old. Binet describes them as follows: “One is calm, reserved, reflective; she pays a great deal of attention to the experiments when she is well disposed. The second is more gay, more exuberant, more giddy, and one must have a great deal of patience to do observations on her” (Pollack and Brenner, 1969, p. 79). In his studies, Binet found strong support for the surprising finding that fluctuations of attention were among the primary predictors of performance in tasks as diverse as length discrimination, number comparison, and color naming: “[…] failure of attention […] is so important that it is always necessary to suspect it when one obtains a negative result. One must then suspend the experiments and take them up at a more favorable moment, restarting them 10 times, 20 times, with great patience. Children, in fact, are often little disposed to pay attention to experiments which are not entertaining, and it is useless to hope that one can make them more attentive by threatening them with punishment” (Pollack and Brenner, 1969, p. 81).

By numerous metrics, today's children are exposed to an environment that challenges attention systems in an even stronger fashion than in Binet's days. Overall, daily information consumption by the USA population as a whole has grown at an average annual rate of 4.4% since 1980, from 9.8 to 33.8 GB/day (Bohn and Short, 2009). This information is consumed at higher rates than in the past. With audiovisual media (television, movies, computer games) leading the way by a large margin, data flow is delivered at an average of 6.4 Mb/s, compared to 2.9 Mb/s in 1980 (Bohn and Short, 2009). Increasingly, leisure and learning activities of children and adolescents are dominated by interactions with media platforms, subjecting them to large amounts of information presented at great density (Skoric et al., 2009). This is increasingly true for children at young ages. As early as 2003, children 6 years and under were reported to spend more than 2 h a day with screen media; about two-thirds grow up in homes where television is on half the time or more, even if no one is watching (Rideout et al., 2003). Managing the exposure to such data flow along with the regulation of its amount, depth, and content has become a formidable challenge for parents and educators. In addition, the ability of an individual to manage limited attentional capacity, e.g., by suppressing irrelevant or distracting dimensions and focusing on the pertinent input, represents an asset of growing importance. Here, we review studies that highlight interindividual differences in the temporal dynamics of ignoring and attending of stimuli embedded in a rapid stream, and their relation with age and cognitive/academic skills. First, we consider the foundations of temporally sustained attentional allocation with an added emphasis on the impact of emotionally driven activation. Next, we describe the developmental trajectory of temporal **attention control** from kindergarten to young adulthood. The final section discusses the association of attention control over time with the development of higher-order cognitive skills and academic competency.

## Attending to Targets Embedded in Rapid Streams: Managing Attention Over Time

Natural environments provide a rich information flow reaching more than only one of our sensory channels at once. This poses a particular challenge for an observer endowed with limited processing capacity. To select the relevant input and respond appropriately, it is necessary to systematically engage and disengage one's attention to pertinent events while ignoring competing stimuli. These skills are intrinsically tied to adaptive behavior and as such paving the way for effective learning inside and outside the classroom. Theoretical and empirical progress in experimental psychology and the cognitive neurosciences has opened avenues for examining the many facets of attentional selection in the context of increasingly complex tasks in a controlled setting. Of particular interest are research designs that require participants to cope with multiple attended objects claiming the same cognitive resources or to manage the temporal competition among relevant and irrelevant items (i.e., targets and distractors). The **rapid serial visual presentation (RSVP)** paradigm is one important approach to probing the temporal dynamics of attention control. In RSVP experiments, stimuli (words, digits, symbols, etc.) are delivered sequentially at a high rate, usually around 10 items per second (see Raymond et al., 1992). Participants are required to search the stimulus stream for target items identified by a specific feature, for instance, a certain shape or color. Thus, the RSVP design shares important aspects with audiovisual media, including television, movies, and computer/console games: Temporal information density is high, and relevant information alternates with irrelevant content.

**Figure 1:**
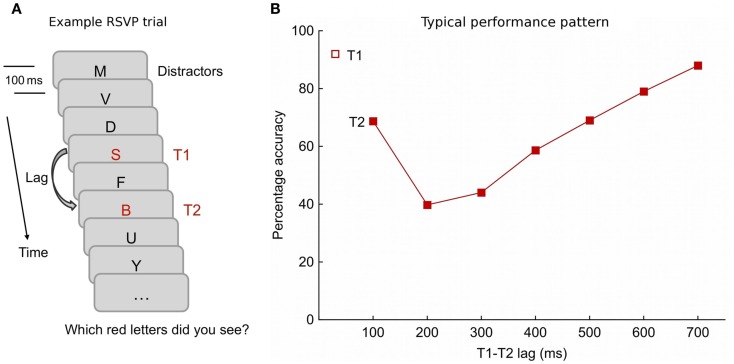
**(A)** Schematic of a dual-task rapid serial visual presentation (RSVP) design that typically results in an attentional blink (AB) effect, i.e., an AB task. Each trial contains a baseline period of distractors, varying in number, before the first target (T1) is presented. The second target (T2) is followed by another series of distractors. The number of intervening distractors between T1 and T2 is manipulated to yield different lag intervals. Stimuli are delivered rapidly usually with an onset-to-onset gap around 100 ms. In this version, participants are to report which red letters they had seen at the end of the trial. **(B)** A hook-shaped performance pattern in the AB task. The profile displays the accurate T2 report given correct T1 identification as a function of lag times. Observers particularly often miss T2 if it falls in a lag window between 200 and 300 ms after T1, but dual target report improves at longer lags. In many instances, superior accuracy translates to the earliest lag (here, 100 ms), where T1 and T2 follow each other immediately. T1 report is shown as a single open square and is usually close to ceiling across lags.

A frequently used variant of the RSVP design involves its implementation as a dual-task, requiring the participant to work on two distinct task demands simultaneously or in rapid succession. A typical trial in a dual-task RSVP paradigm, as illustrated in Figure 1A, invites the report of two highlighted target stimuli (the task doublet) occurring amidst a series of distractors. The number of distractors between the first and second targets (T1 and T2, respectively) is varied to yield different lag times. Given a 10-item per second stimulation rate, zero intervening distractors result in a temporal separation of 100 ms, while six intervening exemplars lead to a lag of 700 ms. A participant's performance profile often follows a hook-shaped path, with lowest report accuracy when T1 and T2 are separated by at least one distractor and about 200 ms of time (see Figure 1B). This performance decrement is referred to as the **attentional blink (AB)**.

The exact mechanism underlying the AB effect has been extensively debated. Many theoretical accounts of the AB emphasize limited capacity, with a potential locus of the “blink” in working memory (e.g., Chun and Potter, 1995; Jolicoeur et al., 2006). Other authors have highlighted the role of attention interference created by the need to rapidly engage and disengage attention, as targets and distractors rapidly alternate (e.g., Di Lollo et al., 2005). This notion has received solid empirical support, highlighting the role of temporal attention selection in the AB (Vul et al., 2008; Nieuwenstein et al., 2009). For instance, attenuated AB impairment is found when participants view multiple targets in a row, without intervening distractors (Di Lollo et al., 2005; Olivers et al., 2007). Furthermore, blink deficits are particularly pronounced when attention is overzealously deployed to the T1, and ameliorated when participants are prevented from over-attending T1, e.g., by listening to music (Olivers and Nieuwenhuis, 2005). Although an in-depth review of empirical findings and theories of the AB is outside the scope of this review, findings suggest that a pure resource-oriented account may have difficulty explaining the entirety of data (Martens and Wyble, 2010). In line with behavioral results, neuroscience research on the AB has consistently shown that trials with missed T2s are characterized by greater brain response amplitudes to T1 and smaller amplitudes to T2, compared to correct T2 trials (Kranczioch et al., 2007; Keil and Heim, 2009). Although consistent with overzealous attention deployment to T1 (Olivers et al., 2007), such effects can also be interpreted in the context of resource sharing accounts of visual processing (Shapiro et al., 2006), or may be taken to indicate that the AB reflects implementation of a cognitive strategy (Wyble et al., 2009). Thus, a variety of factors affecting attention control and stimulus processing can be studied using AB-RSVP paradigms. In addition, since the AB effect has been reliably demonstrated even with very simple stimulus materials, it represents a promising research design for developmental studies of attention.

When discussing the dynamics of attention deployment from a developmental perspective, it is useful to consider that the broad construct of attention is best regarded as a multi-faceted set of processes. Following Luck and Gold (2008), one way to organize processes involved in attentional selection is to distinguish between the control and the implementation of attention. In their view, the concept of control of attention refers to processes responsible for identifying the to-be-selected information, and shielding it against distractors. Neuroanatomically, higher-order cortices (parietal, frontal) are often regarded as the substrate of attention control, because they are capable of sending top-down signals, which may bias sensory processing in favor of attended information (Yantis, 2008). By contrast, implementation processes may be regarded as modes of sensory function that include heightened firing rates of sensory neurons representing attended stimulus locations or features (e.g., color and shape). In the next section, we will focus on the role of attention control as it fluctuates over time, during competition with salient distractors.

## Resisting Salient and Emotionally Arousing Distractors

More than 100 years after Binet's intuitions on motivation and attention, an impressive body of work has demonstrated that emotional activation mobilizes and guides attentional resources to stimuli signaling threat or reward (this is often referred to as **motivated attention**; Lang et al., 1997). These findings are in line with notions of how attention systems assist in the survival of the individual and the species by facilitating detection and identification of essential information. In experimental settings, the effect of emotional content on performance is dependent on the functional role of the affective cue in a given task: Whereas emotional connotation is beneficial for processing a target event, emotionally engaging stimuli are highly potent distractors. Considering the former (benefit) scenario, a series of AB studies have demonstrated that typically developing adults were more likely to report both targets in the critical blink period, when emotionally intense or arousing stimuli served as T2 (e.g., Anderson and Phelps, 2001; Keil and Ihssen, 2004; Anderson, 2005; Keil et al., 2006). As noted by Keil and Ihssen (2004), arousing pleasant and unpleasant words (e.g., to fall in love, to rape) yielded about 15% higher identification scores than neutral exemplars (e.g., to label), during the critical AB period.

Performance in the AB task, however, can be impaired when distractor stimuli rather than targets convey emotionally arousing information. For instance, Most et al. (2005) drew on the finding that an AB may also manifest, when participants search for a simple photograph embedded in a stream of task-irrelevant distractors. The authors placed pictures showing aversive or neutral content (the irrelevant distractor) amidst a stream of upright landscape/architecture images. Adult participants detected the single target (a 90° rotated landscape/architecture photo) less often when an aversive, compared to a neutral, distractor preceded it within a 200-ms time window. These findings indicate that emotionally charged task-irrelevant stimuli may act to attract a disproportionate amount of shared resources, and thus interfere with the performance in the primary task. A number of studies focusing on high-anxious children and their processing bias toward threat-related stimuli have supported this notion. This work has capitalized on the emotional Stroop effect, in which a relative response delay is observed in the font color naming of a word, when the word contains threatening, compared to neutral, information. The delay tends to be more pronounced in anxious children compared to their non-anxious peers (e.g., Vasey and MacLeod, 2001). In terms of developmental trajectories, Kindt and Van Den Hout (2001) suggested that the tendency to prioritize emotionally threatening information is present in younger children, but can be increasingly inhibited as a child grows into early adolescence. Anxious individuals, on the other hand, may fail to learn this skill. Kindt et al. (2000) reported that a bias for spider-related words (e.g., cobweb, hairy) in the Stroop paradigm was present in both fearful and non-fearful 8-year-olds. Around 11 years of age, however, the priority given to threat-related information was decreased in the control group, only.

For the present review, the temporal dynamics of these interference effects are of particular interest. In adults, impacts of emotional distraction on subsequent cognitive function have been found to last across several hundreds of milliseconds (Müller et al., 2008). Addressing the same question, Ihssen et al. (2007) showed university students pictures depicting emotional or neutral scenes (the distractors) 80, 200, or 440 ms prior to a lexical decision task. In the task, participants were asked to judge as quickly and as accurately as possible whether a target string of letters (either a valid neutral verb or verb-like pseudoword) denoted a word or a non-word. Regardless of the pleasure category (e.g., appetitive romance versus aversive attack scenes) and the distractor-target interval, affectively arousing pictures delayed response times to word stimuli. Concurrently recorded electroencephalogram data revealed that an early brain wave evoked by words or pseudowords was reduced subsequent to emotional information. This suggests that electrocortical processing of the targets was relatively impaired when observers were distracted with affective, compared to neutral pictures. Paralleling behavioral effects, suppression of a late brain wave was more pronounced for word than pseudoword targets. Considering the functional roles of the two evoked brain waves, the early effect was assumed to mark final stages of the target analysis, including lexical access, and to mediate the transition from surface analysis to content processing. The late response was interpreted to index word processing on a postlexical level, involving the formation of semantic associations and processes linked to decision making and response planning. Ihssen and colleagues concluded that the presence of prioritized semantic information (i.e., the emotional content of the distracting pictures) interferes with processing lexico-semantic content of target words.

The paradigm outlined above represents a laboratory approximation of situations in which distraction is rapidly followed by cognitive demands and vice versa. Computer users often encounter such scenario when trying to ignore pop-up windows, while searching the World Wide Web for a piece of information. The ability to re-engage attention to a task at hand is also important in the classroom, when students interact with course material that contains, for instance, dry facts as well as appealing cartoons. Using the behavioral design implemented in the Ihssen et al. (2007) study, we have explored temporally extended interference effects in children and describe these findings in Section “Development of Attention Control Over Time,” below.

Taken together, the presence of salient distractors, ubiquitous in a media-driven world, puts an increased load on attention control systems. The negative effects of distraction on performance and brain activity are extended in time, lasting for several hundreds of milliseconds, even after the distractor has disappeared.

## Development of Attention Control Over Time

The early observations of Binet imply that young children experience great difficulty in focusing on a relevant event while ignoring competing information. An excerpt from his description of a little boy reads as follows: “He is apt to forget what he was engaged in doing, to become disgusted with his occupations, or to become distracted by a fantasy, a caprice, an idea which crosses his mind. […] Observe his lack of direction as he goes to school. He does not go straight to the goal as an adult would, but zigzags along, forever stopping or making unnecessary detours to view some spectacle which interests him, distracting him from his goal […]. When absorbed by some occupation, he loses sight of others, and often needs to be told ‘Pay attention’” (Binet, 1909/1975, p. 92, cited in Siegler, 1992). Indeed research dating back to the 1960s has provided valuable insights that the ability to select stimulus input and exert attention control continues to develop from childhood into early adulthood (e.g., Ridderinkhof and van der Stelt, 2000), despite the finding that rudimentary aspects of attention are already present in infancy (e.g., Richards et al., 2010). In an initial study, Strutt et al. (1975) examined the speed at which children (aged 6, 9, and 12 years) and young adults (aged 19 years) could sort cards when one attribute was relevant (e.g., the form of a stimulus), and zero, one, or two dimensions were irrelevant (e.g., a line within the form and/or a star located outside the form's boundary). Sorting times by child participants were found to slow down as the number of irrelevant features was increased. The magnitude of the susceptibility for distraction declined as a function of age. Compatible findings have been obtained with the Eriksen flanker paradigm (Eriksen and Eriksen, 1974). In this task, observers are asked to detect the direction in which a central target (e.g., an arrow) is pointing, while ignoring the orientation of the flanking arrows (the distractors). Accuracy and speed of responses are typically impaired when the flankers point in opposite directions from the target (incongruent condition) rather than aligned in the same direction (congruent condition). These cost effects have been attributed to selective attention, visual confusion, and response competition (see Ridderinkhof and van der Stelt, 2000). It is plausible that the incongruent condition requires a greater amount of attentional selection and control due to the competition between the different responses associated with the target and distractor direction. Developmentally, distraction by incongruent flankers has been shown to improve from age 4 up to approximately 13 years until around the age of 14, when an adolescent performs as accurately and quickly as an adult participant (Rueda et al., 2004a,b; Ladouceur et al., 2007).

Recently, Heim et al. (2011) examined the dynamic process of attention control over time, using the AB paradigm. Two groups of school children had to identify two green targets embedded in a rapid stream of distractors; one task used non-linguistic symbols and the other used letters/words. The temporal distance or stimulus onset asynchrony (SOA) within a target doublet varied between no intervening distractor (116-ms SOA) up to seven intervening distractors (928-ms SOA). First-grade children (aged 6–7 years) showed a decrease in behavioral accuracy for the two targets with increasing temporal proximity of the target items. Fifth and sixth graders (aged 10–11 years) exhibited a performance profile often observed in adult participants: they were able to quickly allocate their attention to two targets in a row (i.e., optimized attentional capacity). This performance profile was accompanied by reduced report rates when target and distractor events alternated rapidly (i.e., optimized attentional control). Preliminary data from a wider cross-sectional study in our laboratory indicate that the most salient changes in AB profiles occur between first and fourth graders. By the time of entry into secondary education programs (in Germany typically around the ages of 10 or 11 years), the blink pattern remains stable, with slight but constant increases in accuracy across temporal lags into late adolescence.

**Figure 2:**
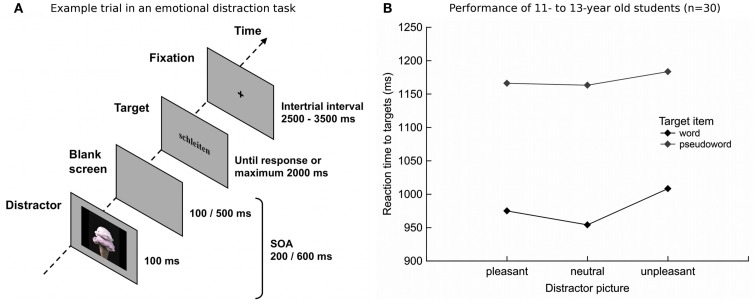
**(A)** Sequence of an example trial in the emotion distraction task. Here, the distractor is an emotional arousing pleasant picture and the target a pseudoword, appearing with a stimulus onset asynchrony (SOA) of either 200 or 600 ms. Early adolescent students were invited to decide as quickly and as accurately as possible whether the target was a real word or a nonsense word by clicking the appropriate mouse button. Each lexical item remained on the screen until the student responded, but no longer than 2000 ms post-target onset. After a variable intertrial interval of 2500–3500 ms and showing a fixation cross, the next sequence started. **(B)** Mean reaction time to verbal targets (in ms) as a function of distractor picture and target item in 30 early adolescents. Emotionally intense (pleasant and unpleasant) distractor images impaired decisions about word, but not pseudoword, targets.

Taken together, these findings suggest that children are endowed with basic forms of attentional control, but that the cognitive capacity and strategic efficiency improves into early adulthood (see also Ridderinkhof and van der Stelt, 2000). The AB paradigm allows researchers to study the time dynamics of attentional engagement and disengagement together in one task, but it has limited ecological validity. By contrast, designs examining the effects of a salient distractor on subsequent cognitive task performance provide greater ecological validity, because they more closely resemble real-world media situations found on television or the World Wide Web. Accordingly, Heim, Ihssen, Hasselhorn, and Keil (under review) sought to provide a quantitative analysis of the extent and timing of emotion distraction on subsequent cognitive demands in early adolescents. In a child-adapted experimental setup implemented previously in adults (Ihssen et al., 2007; see Resisting Salient and Emotionally Arousing Distractors), 11-to-13-year-olds worked on a lexical decision task (the target event) shortly after being briefly presented with task-irrelevant colored pictures (see Figure 2A). Affectively intense photographs (pleasant and unpleasant) impaired processing of subsequent word targets, leading to response speed delays in lexical decision up to about 50 ms, when compared to neutral images (see Figure 2B). Such interference effects emerged irrespective of the temporal distance, showing reaction time slowing for targets presented 200 or 600 ms subsequent to the emotional distractor. Paralleling findings in adults, emotional cues capture and hold shared resources, which are subsequently diminished for processing the target event. Because the longest distractor-target interval in the Ihssen et al. (2007) research amounted only to 440 ms, it is conceivable that the attentional resources of an adult will no longer be captured after longer intervals have elapsed. Pilot data in a sample of 18- to 58-year-old males suggest a reliable, but less prominent response speed delay for word targets (of about 14 ms) at a distractor-target gap as late as 700 ms. This is 2.5 times less than the emotion-induced interference in early adolescents, where the reaction time delay for words was about 35 ms, albeit in the somewhat earlier 600-ms interval. Future studies across different age ranges may systematically manipulate the distractor-target window to examine the temporal pervasiveness of emotional distraction.

## Relation with Academic and Higher-Order Cognitive Skills

Successful acquisition of academic competency, such as in the field of literacy and mathematics, seems to depend to a great extent on the temporal dynamics of cognitive processes. Among other basic skills, a young student must learn to focus his/her cognitive resources on a task-set, while ignoring competing, task-irrelevant, stimuli. This may be particularly difficult when the competing information is more emotionally engaging than the material to be learned. For example, richly illustrated mathematics textbooks featuring cartoons and pictures may invite elementary students to engage with them while at the same time creating heightened demands on attention control. It is conceivable that entertaining artwork attracts attention away from the arithmetic problem that needs to be solved. Such observations open an exciting avenue to systematically study the dynamic processes of attention control in relation to academic education and training. In this vein, a recent review by Stevens and Bavelier (2012) suggests that selective attention skills may play an important role in establishing the neural networks essential for language development and efficient reading. Moreover, some aspects of attention may be altered by remediation and these effects appear to translate into other cognitive domains. For instance, Rueda et al. (2012) reported that 10 sessions of computerized attention training with preschoolers resulted in more adult-like electrical brain responses during a flanker task as well as enhanced abstract reasoning abilities. Conversely, kindergarten children at risk for reading failure benefited from an intensive reading intervention not only in terms of their early literacy skills, but also on measures of brain activity underlying selective auditory attention (Stevens et al., 2011).

**Figure 3:**
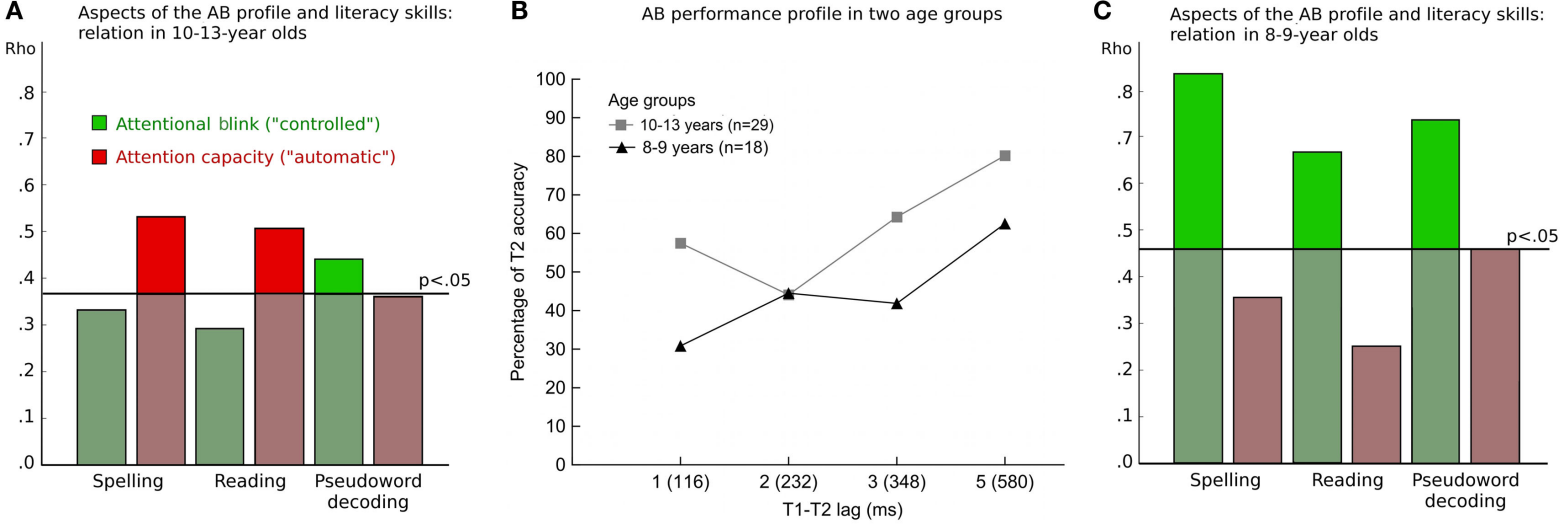
**(A)** Bar plot showing the absolute rank correlation between measures derived from the attentional blink (AB) profile [shown in **(B)**] and indices of literacy in children aged 10–13 years. Literacy scores were based on tests challenging spelling, reading, and pseudoword decoding. Attention profile measures were obtained by calculating (i) the relative decrease at lag 2 yielding in an index of the AB impairment, which characterizes the cost effect of “controlled” attention to the first target (T1), and (ii) the relative decrease at lag 1, where a relatively spared second target (T2) deficit measures the capacity for processing two targets in a row, with no intervening distractors. The horizontal black line indicates the 5% significance level of rank correlation coefficients in this group. **(B)** Percentage of accurate T2 report given T1 identification at four temporal lags of the AB task. Values represent means of 29 older children (gray squares) and 18 younger children (black triangles). **(C)** Bar plot showing the absolute rank correlation between the same measures described in **(A)**, calculated across children in the younger (8–9 years) group. The horizontal black line indicates the 5% significance level of rank correlation coefficients in this sample.

The AB paradigm, allowing researchers to extract various parameters of attention, also represents a promising approach to explore links with indices of cognitive and academic achievement. In a sample of regular elementary students, McLean et al. (2009) reported a positive relation between overall AB performance and reading measures. Skilled readers were better able to identify two targets at a given temporal lag than children with lower reading scores. Focusing on two attentional variants in the AB of 10-to-13-year-olds, Heim et al. (2006) observed differential associations with written language skills: A higher attentional capacity, or the aptitude to quickly focus on two targets in a row, was specifically linked to automatized language processing, including reading/spelling of familiar words. In contrast, enhanced attentional control, or the ability to allocate resources to an initial target, at the cost of processing subsequent events (i.e., the blink), was primarily related to superior performance in controlled language production tasks, such as rule-based decoding of pseudowords (see Figure 3A).

In Section “Development of Attention Control Over Time” we discussed that the AB profile seems to undergo the most prominent changes during the years of elementary education. As illustrated in Figure 3B, the 10-to-13-year-old secondary-school students in the Heim et al. (2006) study already exhibited a hook-shaped performance pattern typically found in adults (e.g., Visser et al., 1999). Second target identification was highest at the longest T1–T2 lag, decreased linearly toward the critical blink interval (lag 2; 232-ms SOA), and showed a relative gain when T1 and T2 occurred within 116 ms in a row. Figure 3B depicts the less mature performance profile of 8-to-9-year-old children attending elementary school (unpublished data). In this group, T2s were subject to more alleviated and temporally diffuse performance impairments for the early lags. Accuracy scores were lowest when T2 followed T1 immediately. Relative to correct reports at the late interval, younger students showed a smaller decrement at lag 2 than the older ones. However, T2 identification remained similarly attenuated at lag 3 (348-ms SOA), when the teenage students exhibited considerable performance sparing. The question arises if attentional variants in the AB pattern of the younger students, who are still in the process of learning to read and spell, show differential links to measures of literacy skills. Indeed, both reading/spelling of age-appropriate words and arbitrary pseudowords were predicted by the ability to specifically allocate attention to one stimulus at a time (see Figure 3C). Less proficient literates seem to benefit from efficient attentional control irrespective of whether the material to be read is new or more familiar. This pattern of results suggests that developmental trajectories for specific sub-processes of selective attention may interact with different stages of academic competency. Across age groups, measures taxing non-verbal intellectual functions, such as visuo-spatial and abstract reasoning skills, did not systematically relate to AB parameters (Heim et al., 2006, 2011). This is in contrast to investigations utilizing the flanker task (e.g., Checa and Rueda, 2011; Rueda et al., 2012). Research on the links between attentional selection and cognitive/academic skills represents a growing area of interest. Future work may address whether and how the various microcomponents of attention regulation differentially relate to general abilities and educational achievement. Consideration of diverse experimental paradigms across various school grades may shed additional light on these findings.

## Synthesis and Perspectives

This focused review argues that a child's ability to control attentional selection over time is a developing skill of crucial relevance to academic performance and adaptive behavior outside the classroom. Of course, attention control is only one specific facet of academic achievement. Children themselves have been shown to be aware that concepts such as desire, intention, and attention are important aspects of successful learning, with this knowledge growing dramatically in early elementary school years (ages 4–6 years; Sobel et al., 2007). Rudimentary strategies of attention control allowing a child to focus on particular sensory inputs can be demonstrated early in development, using a variety of experimental paradigms. By contrast, the more refined ability to manage attentional resources over time in an economic and adaptive fashion appears during early school years, dramatically improves into the early teen years, and continues to develop until late adolescence. Across studies, parameters of attention control over time predict specific aspects of academic performance, rather than general intellectual ability. To the extent that the reported studies are cross-sectional in nature, they cannot examine the plausible hypothesis that different age cohorts, exposed to audiovisual media varying in complexity and speed, may greatly differ in their ability to control temporal selection. For instance, it is a relatively recent phenomenon to see kindergarten-age children equipped with handheld devices capable of presenting complex audiovisual material. Future work may use longitudinal designs to examine potential cohort effects related to these dramatic changes in the sensory-motor environment of children. In the same vein, the sensitivity of specific tasks and distractor types to the developmental trajectory of attention control may be addressed by the systematic manipulation of task, timing, and distractor within the same group of participants, spanning multiple age cohorts. Finally, training programs for children that target specific aspects of attention have recently emerged, aiming to provide remedies for a wide range of problems. These efforts, based partly on experimental work as described above, may in return inform basic science concepts of attention development.

## Conflict of Interest Statement

The authors declare that the research was conducted in the absence of any commercial or financial relationships that could be construed as a potential conflict of interest.

## Key Concept

Attention control: An umbrella term for processes that guide and regulate the way an individual allocates limited capacity to specific sensory events, at the expense of other events. Attention control is particularly relevant when a difficult task is accompanied by numerous potent distractor events.
Rapid serial visual presentation (RSVP): A family of experimental designs in which visual stimuli (letters, numbers, pictures, etc.) are presented to an observer at rapid rates, typically in ranges 5–15 exemplars per second. Participants are asked to detect or identify specific items in this rapid stream, often ascertained by a predefined set of features.
Attentional blink (AB): A transient reduction in report accuracy for the second of two target events embedded in a rapid stream. The AB is most pronounced when the first of two targets is correctly detected/identified and then followed by at least one distractor, before the second target appears. Manipulating the temporal distance between the two targets and the number of intervening distractors, an AB performance profile can be obtained. This profile displays the accuracy in report of the second target as a function of intertarget interval and is often hook-shaped, showing relatively spared performance for conditions in which targets follow each other with no intervening distractor.
Motivated attention: A concept used in the psychophysiology of emotion to describe the fact that sensory systems show facilitation for emotionally arousing stimuli, compared to stimuli that do not engage appetitive or defensive tendencies in the observer. Motivated attention is thought to rely on similar cortical neuro-circuitry as instructed types of attention, in which an individual is prompted to attend to a particular location, feature, or object.
